# 2023. Excess Cost of Healthcare-Associated Infections in the Central Texas Veterans Health Care System

**DOI:** 10.1093/ofid/ofac492.1647

**Published:** 2022-12-15

**Authors:** Richard Nelson, Landon Ashby, Morgan Bennett, Angelia Bridges, Piyali Chatterjee, Hosoon Choi, John David Coppin, Munok Hwang, Thanuri Navarathna, Marjory D Williams, Jing Xu, Chetan Jinadatha

**Affiliations:** University of Utah, Salt Lake City, Utah; Central Texas Veterans Health Care System, Temple, Texas; Central Texas Veterans Health Care System, Temple, Texas; Central Texas Veterans Health Care System, Temple, Texas; Central Texas Veterans Health Care System, Temple, Texas; Central Texas Veterans Health Care System, Temple, Texas; Central Texas Veterans Health Care System, Temple, Texas; Central Texas Veterans Health Care System, Temple, Texas; Central Texas Veterans Health Care System, Temple, Texas; CTVHCS, Temple, Texas; Central Texas Veteran Health Care System, Temple, Texas; Central Texas Veterans Health Care System, Temple, Texas

## Abstract

**Background:**

Many studies reporting the excess cost of healthcare-associated infections (HAIs) have used data from positive cultures found in electronic medical records. These data are often limited in a number of ways including whether the positive culture truly represents an actual infection and not just colonization, when the onset of the infection occurred, and whether the infection was device related. Our objective was to generate estimates of the excess cost of HAIs in one facility, the Central Texas Veterans Health Care System (CTVHCS), using highly reliable information extracted from chart review regarding the infection.

**Methods:**

Our study included data from all inpatient admissions between 1/1/2014 and 12/31/2019 at CTVHCS. HAIs were identified via chart review and were classified based on the infecting organism, specimen location, and whether the infection was device related using 2017 National Health and Safety Network definitions. To minimize time-dependent bias in our estimates of the excess cost of HAIs, we matched each patient with an HAI on day *t* of their inpatient stay to up to 4 patients who had not had an HAI up until day *t* of their inpatient stay. We used multivariable generalized estimating equations models to compare the inpatient costs prior to discharge between patients with and without HAIs. Finally, we used multivariable 2-part models to estimate the impact of HAIs on post-discharge readmission costs.

**Results:**

Our analysis cohort consisted of 425 patients with HAIs who were matched to 1,645 patients without HAIs. HAIs were associated with $29,412 (95% CI:$18,064-$40,759) excess pre-discharge costs, 46.3% increase in the odds of a post-discharge readmission, and $16,049 excess post-discharge costs (see Table 1). The excess pre-discharge costs for central line, catheter-associated urinary tract, and surgical site infections (SSIs) were $96,655 (95% CI:$61,557-$131,754), $34,026 (95% CI:$9,562-$58,491), and $20,014 (95% CI:$5,324-$34,703), respectively. As seen in Table 2, the excess readmission cost for SSIs was $28,222 (95% CI:$9,523-$46,920).

**Table 1:**
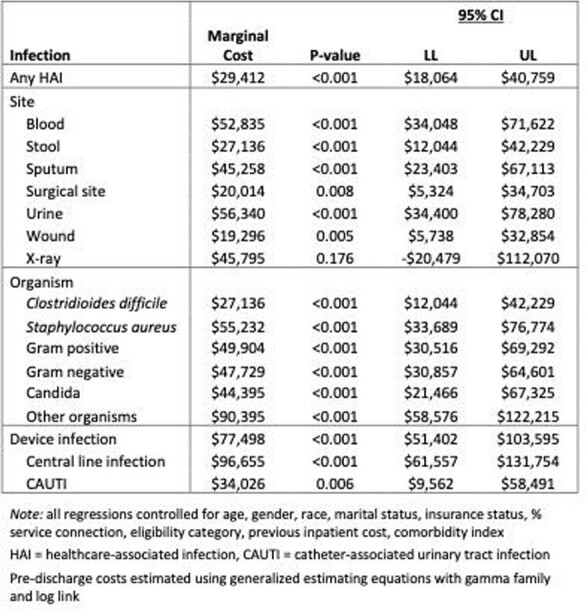
Multivariable regression model results - excess pre-discharge costs associated with healthcare-associated infections

**Table 2:**
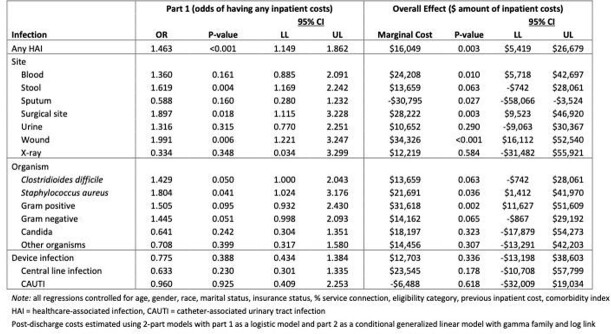
Multivariable regression model results - excess post-discharge costs associated with healthcare-associated infections

**Conclusion:**

We found that HAIs significantly increased both pre- and post-discharge inpatient costs. These costs could potentially be saved if interventions to prevent HAIs could be successfully implemented.

**Disclosures:**

**Piyali Chatterjee, PhD**, AHRQ Grant # 1R03HS027667-01: Grant/Research Support|AHRQ Grant # 1R03HS027667-01: Central Texas Veterans Health Care System **Chetan Jinadatha, MD, MPH**, AHRQ R01 Grant-5R01HS025598: Grant/Research Support|EOS Surfaces: Copper Coupons and materials for testing.

